# Application of Au@Pt Nanozyme as Enhancing Label for the Sensitive Lateral Flow Immunoassay of Okadaic Acid

**DOI:** 10.3390/bios12121137

**Published:** 2022-12-07

**Authors:** Olga D. Hendrickson, Elena A. Zvereva, Vasily G. Panferov, Olga N. Solopova, Anatoly V. Zherdev, Peter G. Sveshnikov, Boris B. Dzantiev

**Affiliations:** 1 Bach Institute of Biochemistry, Research Center of Biotechnology of the Russian Academy of Sciences, Leninsky Prospect 33, 119071 Moscow, Russia; 2 Blokhin National Medical Research Center of Oncology, Ministry of Health of the Russian Federation, Kashirskoye Shosse 24, 115478 Moscow, Russia; 3 Russian Research Center for Molecular Diagnostics and Therapy, Sympheropolsky Blvrd., 8, 117638 Moscow, Russia

**Keywords:** aquatic toxins, okadaic acid, immunochromatography, nanozyme, food safety

## Abstract

In this study, a lateral flow immunoassay (LFIA) was developed to detect okadaic acid (OA) belonging to the diarrheic shellfish poisoning group of aquatic toxins. Newly obtained anti-OA monoclonal antibodies and bimetallic core@shell Au@Pt nanoparticles were used in the indirect format of the LFIA. Peroxidase-mimicking nanozyme properties of Au@Pt enabled using them to enhance band coloration on the test strips and, consequently, for increasing the LFIA sensitivity. The instrumental limit of detection (LOD), the working range of detectable concentrations, and the visual cutoff of the assay were 0.5, 0.8–6.8, and 10 ng/mL, respectively. The assay duration was 20 min. The rapid and simple sample preparation procedure was applied for seawater, river water, and fish samples. The total duration of the sample pretreatment and LFIA was 25/40 min for water/fish samples, ensuring testing rapidity. The developed test system provides sensitive control of raw materials and food products and can be used to detect OA at all stages of the food industry «from sea to fork» chains.

## 1. Introduction

Among the dangerous food contaminants, a special place belongs to aquatic toxins—the metabolites of cyanobacteria and microalgae inhabiting both fresh and saltwater bodies [1,2,3]. The cyanobacteria and microalgae producing cyanotoxins and phycotoxins, respectively, are initial links of food chains. Aquatic toxins may accumulate in different marine or freshwater macroorganisms including mollusks and fish [4,5]. Consumption of contaminated food can cause severe acute poisoning in humans. Regular intake of trace amounts of aquatic toxins leads to long-term chronic disorders, such as hepatotoxicity, oncological diseases, neuro-, immuno-, embryotoxic effects, etc. [6]. Phycotoxin okadaic acid (OA), produced by *Prorocentrum* and *Dinophysis* dinoflagellates, belongs to the priority aquatic toxins [7]. These algae, widespread in the coastal waters of different regions, cause the so-called “red tides” during their active reproduction [4]. The action of lipophilic and heat-resistant OA is expressed in the inhibition of protein phosphatases; because OA poisoning mainly consists of disorders of the gastrointestinal tract, it is classified as a diarrheic shellfish poisoning (DSP) toxin [7,8].

High concern about the pervasion of toxic substances into aquaculture foodstuffs has led to official regulations and monitoring of aquatic toxins in products for human consumption. Thus, the maximum residue level (MRL) established in the EU countries for OA in mollusk tissues is 0.16 μg/g [9]. Initially, biotests on living organisms were used to monitor aquatic toxins [10]. However, due to ethical issues and difficulties with harmonization, biotests have been replaced by instrumental methods. Liquid/gas chromatography-mass spectrometry (LC/GC-MS) is traditionally used to determine aquatic toxins [11,12]. LC/GC-MS is an accurate and sensitive method for the selective identification and quantification of individual toxins and their derivatives. Its disadvantages include expensive and complex instrumentation as well as the thorough sample preparation (most often by solid-phase extraction), which significantly increases the labor and time requirements of the analysis. Therefore, continuous mass monitoring of toxins content in mariculture products by instrumental analytical methods is expensive, difficult, and impractical.

Rapid and sensitive qualitative detection (yes/no) and (semi-)quantitation of aquatic toxins require simple, productive, and low-cost methods that provide information on the presence and level of toxins quickly and with minimal effort. Lateral flow test systems with immune recognition of analytes fully met these requirements [13,14]. Lateral flow immunoassay (LFIA) of OA is described in several studies [15,16,17,18]. All the reported assays are based on the direct LFIA format, where anti-OA specific antibodies are conjugated with gold nanoparticles (AuNPs) as a marker. Among real samples, only the seafood matrix was tested for OA content. Our previous investigations in this field were aimed at multiplex determination of OA, domoic acid, and microcystin-LR as representatives of DSP, amnestic shellfish poisoning toxins, and hepatotoxins, respectively [19,20]. Besides, the enhanced LFIA of OA with cascade signal amplification was implemented [21].

In this study, several clones of anti-OA monoclonal antibodies (MAbs) were produced and tested. Instead of common AuNPs, bimetallic core@shell Au@Pt nanozyme was synthesized and used as a catalytic label with peroxidase-mimic catalytic properties. Au@Pt label was attached to anti-species antibodies to perform the indirect format of the LFIA. In contrast to the direct assay, in the indirect one, the unproductive immune binding without signal change is excluded, which contributes to the assay sensitivity [22]. Among other advantages of the indirect LFIA, the significant saving in specific antibodies and the application of labeled anti-species antibodies as a universal reagent should be emphasized. The developed test system was used to determine OA in the extended panel of samples, including fish and water.

## 2. Materials and Methods

### 2.1. Chemicals and Materials

OA, soybean trypsin inhibitor (STI), bovine serum albumin (BSA), gold(III) chloride hydrate (HAuCl_4_·H_2_O), sodium hexachloroplatinate (IV) hexahydrate (Na_2_PtCl_6_·6H_2_O), sodium ascorbate, methanol, sucrose, dimethyl sulfoxide (DMSO), N-hydroxysuccinimide (NHS), Triton X-100, sodium azide, 3,3′-diaminobenzidine (DAB), hydrogen peroxide (30% solution), and N-(3-dimethyl aminopropyl)-N′-ethyl-carbodiimide hydrochloride (EDC) were from Sigma-Aldrich (Saint Louis, MO, USA). A peroxidase substrate solution based on 3,3′,5,5′-tetramethylbenzidine (TMB) was purchased from Immunotech (Moscow, Russia). Polyclonal goat anti-mouse immunoglobulins (GAMI) were from Arista Biologicals (Allentown, PA, USA), polyclonal donkey anti-goat immunoglobulins (DAGI), and GAMI labeled with horseradish peroxidase (GAMI–HRP) were from Jackson Immuno Research Labs (West Grove, PA, USA).

### 2.2. Transmission Electron Microscopy, Atomic Force Microscopy, and Energy-Dispersive X-ray Spectroscopy

To perform transmission electron microscopy (TEM), 7-µL aliquots of AuNPs or Au@Pt nanozyme solution in distilled water were applied to copper grids (300 mesh) coated by a film of polyvinyl formal dissolved in chloroform and incubated for 15 min. After that, grids were blotted and dried for 12 h at room temperature. TEM images were obtained on a JEM CX-100 electron microscope (Jeol, Tokyo, Japan) at an accelerating voltage of 80 kV and magnifications of 33,000–3,300,000. The digital microphotographs were analyzed using Image Tool software (University of Texas Health Science Center, San Antonio, TX, USA).

To perform atomic force microscopy (AFM), aliquots of AuNPs or Au@Pt nanozyme solutions in distilled water were applied to the surface of freshly-cleaved mica and incubated for 20 min. After that, the mica was blotted and dried for 12 h at room temperature. The measurements were performed using a Smart SPM-100 atomic force microscope (AIST-NT, Dolgoprudny, Russia) and a rectangular-shaped cantilever with a 1nm curvature radius (fpN01HR, AIST-NT, Novato, CA, USA). The measurements were performed in a tapping mode. The root mean square of nanoparticles was calculated as the standard deviation of the heights (z-values) in the nanoparticles cross-section using Gwyddion 2.42 (Czech Metrology Institute, Brno, Czech Republic).

Elemental analysis of Au@Pt nanoparticles was carried out by the energy-dispersive X-ray spectroscopy (EDX) using a Jeol JEM-1400 transmission electron microscope (Jeol, Tokyo, Japan) equipped with an INCA Energy TEM 350 energy-dispersive spectrometer (Oxford Instruments, High Wycombe, UK). For this, 20-µL aliquots of Au@Pt solution in distilled water were applied to copper grids (300 mesh) coated by a film of polyvinyl formal dissolved in chloroform and incubated for 15 min. Then, grids were blotted and dried for 12 h at room temperature.

### 2.3. Synthesis of OA–Protein Conjugates

OA was conjugated with BSA (to obtain a coating antigen) and STI (to obtain an immunogen) based on Wang et al.’s (2017) protocol [16]. For this, EDC (100 μL, 5 mg/mL), NHS (100 μL, 9 mg/mL), and OA (100 μL, 5 mg/mL), all in DMSO, were mixed and shaken for 30 min at room temperature (RT). Then, STI or BSA (400 μL, 5 or 2.5 mg/mL, respectively, in 50 mM carbonate buffer, pH 9.5) was added dropwise and shaken for 2 h at RT. Finally, the reaction mixtures were dialyzed for 16 h at 4 °C against 50 mM K-phosphate buffer, pH 7.4, with 0.1 M sodium chloride (PBS).

### 2.4. Production of MAbs

Anti-OA MAbs were produced using female BALB/c mice (1.5–2 months of age) with OA–STI as an immunogen [23]. Mice were kept under standard laboratory conditions with water and food ad libitum. At the end of the experiment, the animals were euthanized with an intravenous injection of pentobarbital. Animal studies were performed following the EU Directive 2010/63/EU and authorized by the Ethics Committee of the Research Center of Biotechnology (protocol # N22-D dated 12 February 2020).

### 2.5. Enzyme-Linked Immunosorbent Assay of OA

OA–BSA (1 μg/mL, 100 μL in PBS) was adsorbed in the transparent 96-well polystyrene microplates (Costar 9018, Corning Costar, Tewksbury, MA, USA) overnight at 4 °C. Every ELISA stage was accompanied by fourfold washing of the wells with PBS with 0.05% Triton X-100 (PBST). Further, all immunoreagents were diluted in PBST.

To determine MAbs’ titer, all clones of MAbs (53–0.22 μg/mL, 100 μL) were added to the wells and incubated for 1 h at 37 °C. For the detection of OA, OA (167 ng/mL–8.5 pg/mL, 50 μL) and anti-OA MAbs solutions (20 ng/mL for the clones Okad B4, Okad H1, and Okad C2, 60 ng/mL for the clones Okad D6 and Okad A5, 50 μL) were poured. The incubation for 1 h at 37 °C was performed for both assays. Then, the GAMI–HRP conjugate (100 μL, 1:4000 dilution) was added and incubated for 1 h at 37 °C. To measure HRP activity, 100 μL of the TMB substrate solution was added to the wells and incubated for 5–15 min at RT. The reaction was stopped by 1 M H_2_SO_4_ (50 μL per well), and the optical density at 450 nm (OD_450_) was measured by a microplate photometer (Zenyth 3100, Anthos Labtec Instruments, Wals, Austria).

### 2.6. Synthesis of AuNPs, Au@Pt Nanozyme, GAMI–AuNPs, and GAMI–Au@Pt Conjugates

AuNPs were synthesized by the approach described in [24]. To obtain 30-nm/10-nm AuNPs, 1/1 mL of 1% HAuCl_4_·H_2_O was added to 97.5/96 mL of deionized water and heated to a boil. Then, 1.5/3 mL of 1% sodium citrate was added, and the resulting mixture boiled for 20/25 min under reflux. The obtained AuNPs were cooled and stored at 4 °C.

Au@Pt was synthesized as described by Gao et al. (2017) [25]. AuNPs with a diameter of 10 nm (20 mL) were mixed with 10 mM Na_2_PtCl_6_·6H_2_O (4 mL). Then, 5.3 mL of deionized water was added to this mixture. The resultant solution was heated to 80 °C, after which 50 mM sodium ascorbate (4 mL) was added using a peristaltic pump with a flow rate of 450 μL/min. Then, the reaction mixture was kept at 80 °C for 20 min. After cooling, Au@Pt was centrifuged at 12.000× *g* for 20 min. The sediment was resuspended in the deionized water to avoid aggregation in the reaction media having high ionic strength.

To obtain GAMI–AuNPs conjugate, 30-nm AuNPs were used. GAMI at a concentration of 6 μg/mL selected according to [26] was dialyzed against 10 mM Tris-HCl buffer, pH 8.5. Afterward, GAMI were added to the solution of AuNPs having pH 8.5 (previously adjusted by 0.1 M K_2_CO_3_). The mixture was incubated for 1 h with stirring at room temperature, then BSA was added to a final concentration of 0.25% and stirred for another 15 min. The resulting conjugate was precipitated by twice centrifugation at 9200× *g* for 15 min at 4 °C and resuspended in 10 mM Tris-HCl buffer, pH 8.5, containing 1% BSA, 1% sucrose, and 0.05% sodium azide.

To obtain GAMI–Au@Pt conjugate, a solution of Au@Pt nanozyme was first adjusted to pH 8.5 by 0.2 M Na_2_CO_3_. Then, GAMI was added to obtain final concentrations of 2, 4, 6, 8, 10, and 12 μg/mL and incubated for 1 h at RT. Then, a 10% solution of BSA was added to a final concentration of 0.25% and stirred for another 15 min. After complexation, GAMI–Au@Pt conjugates were centrifuged at 12,000× *g* for 20 min. The sediments were resuspended in 10 mM Tris buffer, pH 8.5, containing 1% BSA, 1% sucrose, and 0.1% sodium azide, to concentrate a solution ~15–20 times relative to the original GAMI–Au@Pt solution. 

The obtained conjugates were stored at 4 °C.

### 2.7. Preparation of LFIA Test Strips

Two kinds of test strips were prepared for the LFIA with AuNPs and Au@Pt nanozyme as labels. In both cases, CNPC-SS12 nitrocellulose membrane on plastic support (Advanced Microdevices, Ambala Cantt, India) and ReliaFlow 319 membrane (Ahlstrom-Munksjö, Helsinki, Finland) were applied as working and adsorption membranes, respectively. For AuNPs-based LFIA, a GFB-R4 sample pad (Advanced Microdevices, Ambala Cantt, India) was applied. For Au@Pt-based LFIA, no sample pad was used, and the plastic support was cut to the bottom edge of the working membrane.

For both LFIAs, OA–BSA (0.5 mg/mL in PBS) and DAGI (0.1 mg/mL in PBS) were immobilized on the working membrane with a flowrate of 0.1 μL/mm to obtain a test (T) and a control (C) zone, respectively. The automatic dispenser (Iso-Flow, Imagene Technology, Hanover, NH, USA) was used for this purpose. After overnight drying of the multimembrane composites at RT and for 2.5 h at 37 °C, test strips of 2.9 mm width were prepared using an automatic guillotine (KinBio, Shanghai, China). Test strips were stored in sealed packages with silica gel at RT.

### 2.8. Sample Preparation of Seawater and Fish

Seawater samples were collected from the Barents Sea (Murmansk, Russia) and the Aegean Sea (Fethiye, Turkey). The river water sample was from the Volkhov river (Velikiy Novgorod, Russia). All water samples were stored at 4 °C. Before the LFIA, Triton X-100 was added to them to its final 0.05% concentration. Then, the mixtures were spiked with OA and 10-fold diluted by PBST.

Fish (trout) purchased in a local food store was minced using a blender. To a 0.5 g sample, OA (60 μL, 10 μg/mL, which corresponds to 1.2 µg/g), and the methanol-water (1:1) mixture (5 mL) were added. The obtained preparation was stirred for 5 min and then centrifuged (1500× *g*, 10 min). The supernatant was 10-fold diluted by PBST before the LFIA.

### 2.9. Standard and Enhanced LFIAs of OA

For the detection of OA, its solutions (1000–0.05 ng/mL, 50 μL in PBST), anti-OA MAbs (450 ng/mL and 112.5 ng/mL for the standard LFIAs with AuNPs and Au@Pt nanozyme, respectively, and 45 ng/mL for the enhanced assay with Au@Pt nanozyme, 50 μL in PBST) were mixed. Then, GAMI–AuNPs (4 μL) or GAMI–Au@Pt (2.5 μL) were added to the mixtures and incubated for 3 min at RT. After that, the test strips were immersed in the mixtures and incubated for 15 min.

In the standard LFIAs with AuNPs and Au@Pt nanozyme, the test strips were taken out of solutions, blotted, and scanned as is using a CanoScan LiDE 90 scanner (Canon, Tokyo, Japan). In the enhanced LFIA with Au@Pt nanozyme, the test strips were taken out, put horizontally, and blotted. DAB-based substrate solution (125 μL of 1% DAB and 125 μL of 0.3% hydrogen peroxide in distilled water were mixed with 2.5 mL of PBS, pH 7.2, and used immediately after preparation) was applied to test strips (25 μL per each strip) and incubated for 2 min. Then, test strips were scanned as described above. The coloration of T and C zones was assessed by the TotalLab software from Nonlinear Dynamics (Newcastle upon Tyne, Great Britain).

### 2.10. Evaluation of the LFIA and ELISA Results

To build the plots of color intensity (in relative units, RU) or OD (y) versus the analyte concentrations (x) and fit them using a four-parameter logistic function, Origin software from OriginLab (Northampton, MA, USA) was applied. The LODs, working ranges, and cutoffs were determined in accordance with Uhrovcik (2014) [27].

## 3. Results and Discussion

### 3.1. Obtaining the Immunoreagents

As the OA-binding reactant, MAbs were produced using common hybridoma technology. The conjugate of OA with STI synthesized by carbodiimide activation was used as an immunogen. As a result, 5 clones of MAbs were obtained: Okad H1, Okad B4, Okad C2, Okad D6, and Okad A5. Their preliminary screening was performed by enzyme-linked immunosorbent assay (ELISA). MAbs were characterized by titers (the minimum concentration of antibodies that provides a reliably detectable interaction with the immobilized antigen). In addition, LODs of OA were determined in the ELISA using each of the produced antibody clones. One of the obtained clones (Okad A5) demonstrated no competitive effect during the ELISA, which can probably be explained by the recognition of the spacer region between the hapten and the protein carrier by this clone of MAbs. That’s why it was excluded from the list of potential reagents for the test system. For other clones, the dependences of OD on MAbs’ concentration and calibration curves of OA in the ELISA after immobilization of various protein conjugates of OA are given in Figures S1 and S2. Titers and LODs are shown in Table 1.

As can be seen, the minimum LOD of OA was achieved using the Okad D6 clone (0.2 and 0.9 ng/mL with OA–BSA and OA–STI immobilization, respectively). It should be noted that no interaction between all clones of MAbs and the immobilized STI and BSA was observed, which indicated that MAbs had specificity towards the hapten and not to the protein component of the immunogen. Because the MAbs titer upon the OA–BSA immobilization was slightly lower than that upon the OA–STI immobilization, the former conjugate was selected for the LFIA.

**Figure 1:**
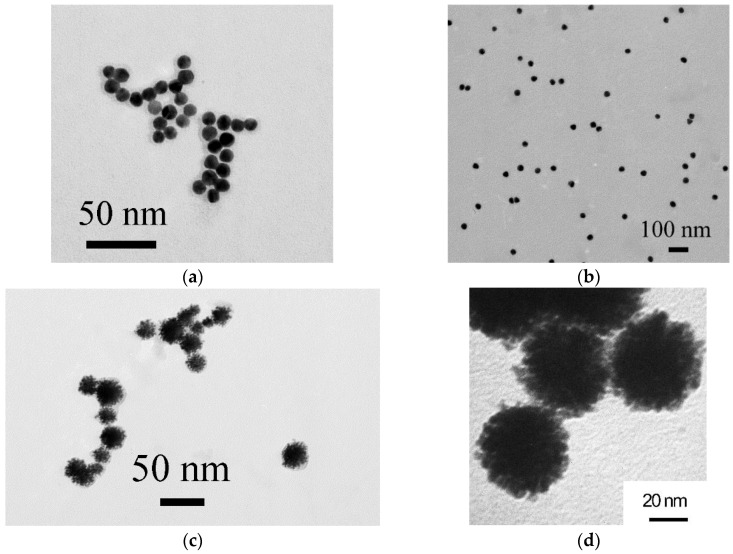
Microphotographs of smaller (**a**) and larger (**b**) AuNPs, and Au@Pt nanozyme (**c**,**d**).

AuNPs with different dimensional characteristics were obtained by reducing gold salt with sodium citrate. Larger and smaller AuNPs can be synthesized by varying such reaction conditions as the concentrations of HAuCl_4_ and the reducing agent and the reaction time. The small-sized AuNPs were intended as cores for Au@Pt core@shell nanozyme, and the larger AuNPs as a traditional colorimetric label [28]. AuNPs were characterized by TEM. Both preparations contained nonaggregated particles homogeneous in size and shape with an average diameter/ellipticity of 10.4 ± 1.5 nm/1.1 ± 0.1 and 27 ± 2 nm/1.14 ± 0.1, respectively (Figure 1a,b).

The nanodispersed Au@Pt core@shell nanozyme, having peroxidase-mimicking activity, was also used as a marker in the lateral flow test system. The nanozyme was obtained based on small AuNPs by reducing a platinum salt with sodium ascorbate on a gold surface [25]. Due to its intense black color, the nanozyme can be used as a direct colorimetric marker in the standard LFIA. To exploit the catalytic properties of the label, a peroxidase substrate has to be added at the final stage of the LFIA (enhanced assay). It should be noted that the catalytic activity of nanozymes is primarily determined by the surface area; their activity increases with decreasing particle size [29]. In addition, particles with a branched surface have higher catalytic activity than spherical nanozymes [30]. Considering these features, gold-cored and platinum-shelled particles were obtained. According to TEM, the obtained particles had a diameter of approximately 15–25 nm (Figure 1c). The detailed study of the structure and surface morphology was carried out by TEM under high magnification (3,300,000). The nanozyme was characterized by an urchin-shaped morphology (Figure 1d).

**Figure 2:**
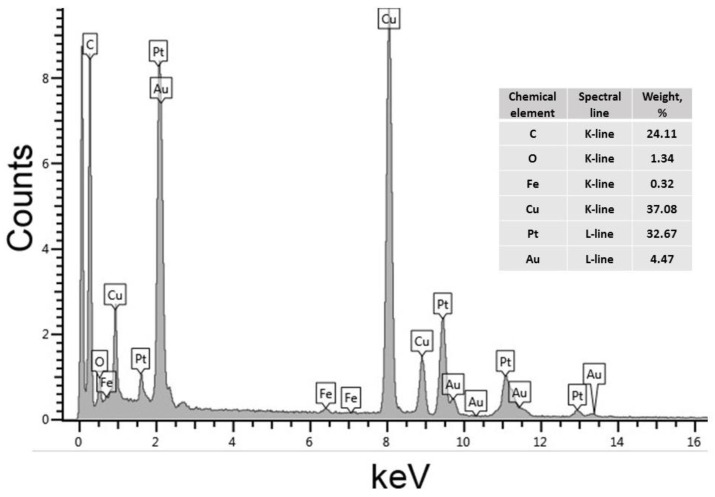
EDX spectrum of Au@Pt nanozyme and its elemental composition.

To study the composition of the Au@Pt nanozyme, its elemental analysis was carried out using EDX. The obtained EDX spectrum and its analysis in terms of the nanoparticles’ composition are presented in Figure 2.

The spectrum contains peaks characteristic of Au and Pt, which confirms the formation of nanoparticles with these elements in their composition. The detected peaks of carbon, oxygen, copper, and iron arise from the polyvinyl formal-coated copper grids used to support the analyzed sample and a sample holder applied during the spectroscopy. The formation of nanostructures having a core@shell morphology was early demonstrated by Gao et al., (2017) (whose protocol was used in this study to synthesize a nanozyme (see Section 2.6)) by EDX mapping [25]. The formation of core@shell Au@Pt nanoparticles was expected because the standard electron potential of the PtCl_6_^2−^/Pt pair (0.74 V vs. the standard hydrogen electrode) [31] is lower than that of Au/AuCl_4_^−^ (0.99 V vs. the standard hydrogen electrode) [32]. Therefore, Pt cannot be deposited on AuNPs by galvanic replacement. In the presence of sodium ascorbate as a reducing agent, Pt is deposited around AuNPs, which was confirmed by the EDX spectrum (Figure 2).

The formation of the Pt shell follows the Volmer-Weber growth mode: the platinum part of the nanoparticles grows as separate islands on a gold surface rather than a smooth coating [33]. The non-uniform distribution of Pt was confirmed by measuring the roughness of Au@Pt nanoparticles by AFM. The mean square for bare AuNPs was 0.4 ± 0.1 nm (*n* = 20) and for Au@Pt—1.7 ± 0.5 (*n* = 15) (Figure S3a,b). As a complementary method to confirm the formation of a Pt shell around the Au core, dynamic light scattering (DLS) of Au@Pt nanoparticles generated after adding different amounts of Pt salt was registered. It was shown that upon an increase of PtCl6^2−^concentration, the hydrodynamic diameter of the formed Au@Pt nanoparticles steadily increased (Figure S4).

To implement the OA LFIA in the indirect competitive format, conjugates of markers (AuNPs and Au@Pt) with anti-species antibodies (GAMI) were obtained. For the AuNPs-based label, the conditions of the antibodies’ immobilization were chosen using the flocculation curve (the dependence of the OD of the gold solution on the concentration of the immobilized protein) based on ensuring the stability of the conjugates [34]. According to our previous studies, such surface stability was guaranteed if GAMI were immobilized at a concentration of 6 μg/mL [26].

A load of antibodies on the nanozyme label was chosen taking two requirements into account. On the one hand, the chosen concentration of GAMI should provide an adequate level of binding in the T zone. On the other hand, it should not interfere with the catalytic reaction on the surface of the nanozyme, shielding the catalytic surface of gold. For the synthesis of GAMI–Au@Pt conjugates, antibody loading was carried out in the range of 2–12 μg/mL. The antibodies were immobilized by physical adsorption. The resulting conjugates were designated as GAMI–Au@Pt2, GAMI–Au@Pt4, GAMI–Au@Pt6, GAMI–Au@Pt8, GAMI–Au@Pt10, and GAMI-Au@Pt12.

### 3.2. Standard LFIAs of OA Using AuNPs and Au@Pt Nanozyme as Labels

The first stage of this study was the implementation of the standard LFIA with AuNPs having a diameter of about 30 nm, which are commonly used as a red-colored marker in the lateral flow test systems for the determination of various analytes [28]. Hereafter, the term “standard LFIA” means the analysis based on detecting a coloration provided by a marker itself without additional manipulations on signal amplification. The standard LFIA was performed in an indirect competitive format. The tested sample was mixed with anti-OA MAbs and labeled anti-species antibodies (GAMI–AuNPs). As a result, a MAbs–GAMI–AuNPs complex was formed, the movement of which along the membrane was initiated by the contact of the test strip with the liquid reaction mixture. Specific antibodies of this complex interacted with the OA–BSA conjugate immobilized in the T zone, which was accompanied by the formation of a lower red line on the test strip. The excess of the complex moved to the C zone, bound there with other anti-species antibodies (DAGI) with the formation of an upper red line. If the sample contained OA, the latter interacted with specific MAbs (to form the OA–MAbs–GAMI–AuNPs complex), inhibiting the label binding with the immobilized OA–BSA conjugate. Thus, the intensity of the coloration in the T zone inversely depends on the concentration of OA in the sample. 

The calibration curve of OA determination by the standard LFIA with AuNPs is presented in Figure S5. The LOD (instrumental)/cutoff (visual LOD) of OA was 1.2/20 ng/mL; the working range of detectable concentrations was 2.5–26.8 ng/mL.

**Figure 3:**
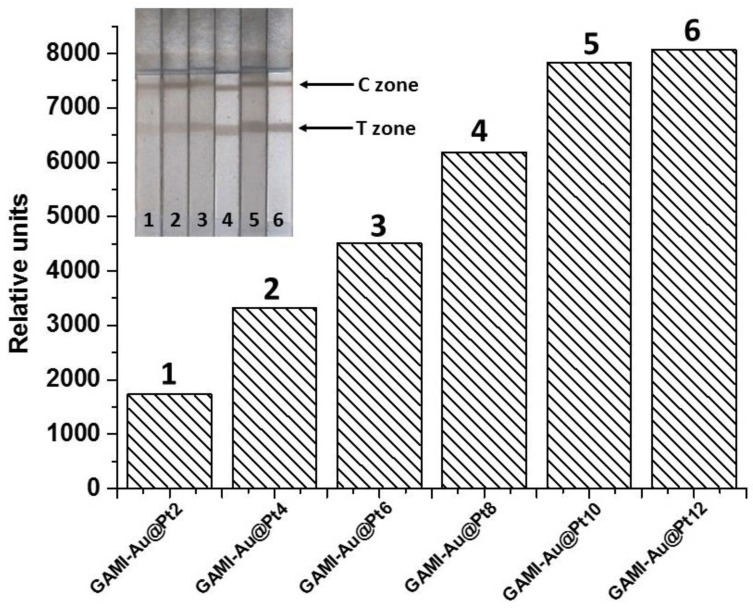
The dependence of coloration intensity in the T zone of the test strips on the loading of GAMI in the conjugates in the standard LFIA of OA with Au@Pt nanozyme and the images of the test strips. In the control experiment (GAMI loading is 0 µg/mL), there is no analytical signal in the T zone.

The standard LFIA with Au@Pt label was performed in the same format as described above, except that GAMI–Au@Pt was used instead of GAMI–AuNPs, and the black-colored bands appeared in the T and C zones of test strips. Before the competitive detection of OA, the GAMI–Au@Pt conjugate was selected from the obtained panel. For this, the dependence of the coloration intensity in the T zone of the test strip on the GAMI content in the conjugates was studied. The experiment was performed at MAbs’ concentration of 300 ng/mL and an amount of all GAMI–Au@Pt conjugates in the reaction mixture of 2 µL. Testing was carried out in two stages: first, specific MAbs and the GAMI–Au@Pt conjugate were incubated, and then, the test strips were immersed in the reaction mixture. Initially, the LFIA was carried out using test strips in a composition that included a sample pad. It was shown that GAMI–Au@Pt conjugate was partially retained on the sample pad, which required a higher consumption of the label and an assay time of ~30 min to ensure the intense coloration of test strip zones. Therefore, the test strips were cut under the lower edge of the working membrane. This, firstly, decreased the sample volume (from 100 µL for a full-size test strip to 50 µL for a cut one) and the amount of the added label, and, secondly, reduced the assay time. The dependence of the T zone coloration intensity on the load of GAMI in the conjugates is shown in Figure 3.

**Figure 4:**
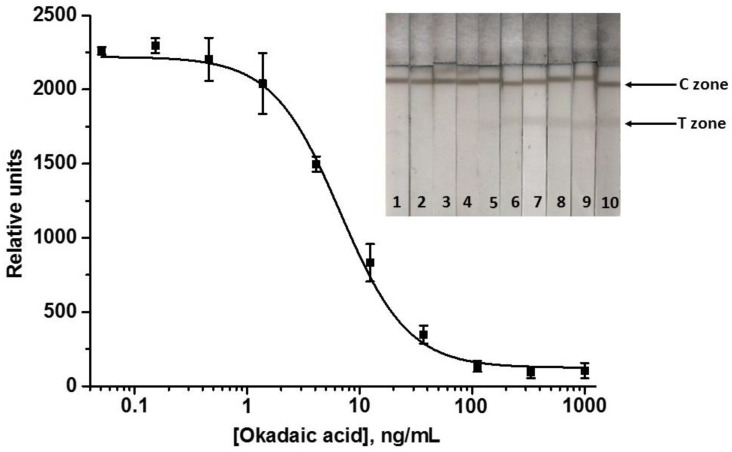
Calibration curve of OA in the standard LFIA with Au@Pt nanozyme and images of the test strips. The following OA concentrations were detected (ng/mL): 1000 (1), 333 (2), 111 (3), 37 (4), 12.3 (5), 4.1 (6), 1.4 (7), 0.45 (8), 0.15 (9), 0.05 (10) (*n* = 3).

The figure shows that the coloration intensity in the T zone increases with the increase of GAMI loading from 2 to 12 µg/mL. For the OA LFIA, the GAMI–Au@Pt6 conjugate was chosen, which provided an average intensity in the studied range (~4500 RU). This choice can be explained by the fact that upon a further decrease in MAbs’ concentration (required to reduce the OA LOD), this marker conjugate will provide some margin of signal intensity, which will also be expected to decrease upon such action. LFIA parameters (concentrations of free and immobilized reagents and duration of the assay steps) were optimized to achieve the lowest LOD with a signal amplitude enough for reproducible and accurate results. The concentration of the immobilized OA–BSA was varied in the range of 0.1–0.75 ng/mL; the concentration of the immobilized DAGI was varied from 0.05 to 0.2 mg/mL. The volume of the added label was 1.5–4 µL per strip. The concentration of the added MAbs was reduced by more than 2 times (from 300 to 112.5 ng/mL). The time of the first LFIA stage varied from 2 to 5 min, and the second stage from 10 to 30 min. Experiments have shown that the requirements mentioned above were met upon the following parameters: 0.5 mg/mL of OA–BSA, 0.1 mg/mL of DAGI, 2.5 µL of GAMI–Au@Pt6, 3-min preincubation, and 15-min incubation with the test strip. The maximum amplitude of the colorimetric signal was ~2250 RU. A further decrease in the concentrations of specific MAbs led to the essential decrease in the signal intensity negatively affecting the assay accuracy. The resultant calibration curve of OA and images of the scanned test strips are presented in Figure 4.

The standard LFIA with Au@Pt nanozyme allowed for the determination of OA with the LOD of 1.5 ng/mL and a working range of detectable concentrations of 2.6–6.7 ng/mL. The cutoff was 20 ng/mL. As can be seen, the analytical parameters of the standard Au@Pt-based LFIA do not significantly differ from those estimated for AuNPs-based LFIA.

### 3.3. Enhanced LFIA of OA

**Figure 5:**
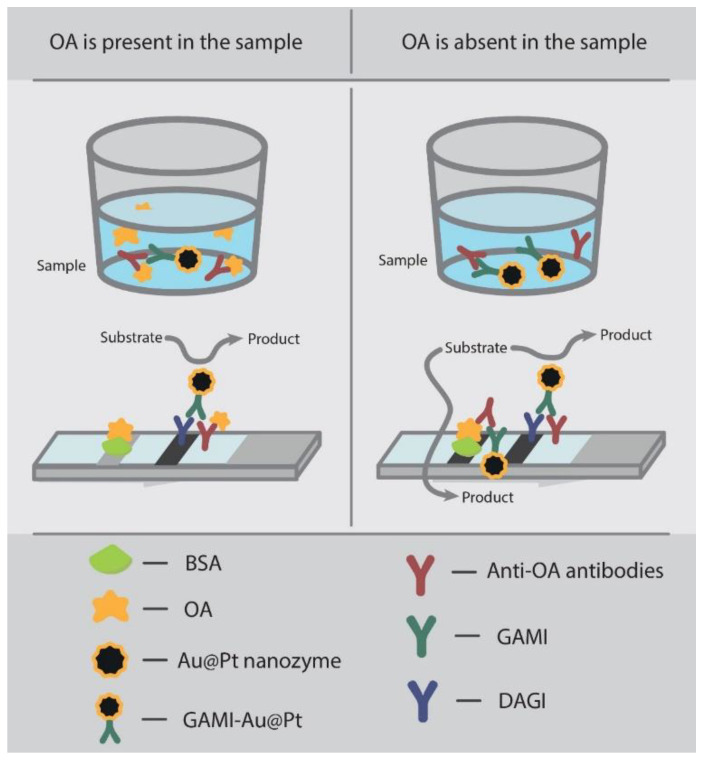
Scheme of the enhanced LFIA of OA using Au@Pt nanozyme.

The enhanced LFIA was based on the catalytic properties of the nanozyme label. It was implemented as described above, except that peroxidase substrate was applied to the membrane after the LFIA. The scheme of the assay is shown in Figure 5.

The Au@Pt nanozyme catalyzes the transformation of the substrate into a colored product, which increases the coloration intensity of test strip zones. An important part of the assay development was the choice of the substrate mixture. It was necessary to choose a substrate that forms an insoluble precipitate adsorbed on the T and C zones of the working membrane. Several compositions of substrate mixtures based on TMB and DAB as the most commonly used chromogenic peroxidase substrates were tested; their detailed composition is presented in Table S1.

TMB substrate, which is popular for the ELISA with the HRP label [35], including the OA ELISA described in this study, led to the appearance of the coloration in the zero point (with no anti-OA MAbs in the tested sample). Because of its non-specific background, it was excluded from the list of applicable substrates. For DAB-based substrates, this drawback was absent. Nine compositions of the DAB substrate were tested (Table S1, solutions 2–9). All of them contained DAB and hydrogen peroxide at different ratios and, in some cases, cobalt or nickel chlorides, which are usually added to the DAB substrate to enhance the coloration of the product of the catalytic reaction [36]. All substrates were converted to insoluble brown-colored precipitates. Finally, it was shown that metal salt additives contributed not only to the intensity of the bands in the T and C zones but also to the uneven background coloration of the whole working membrane. Therefore, the increase in coloration of the zones was compensated by a high and uneven background signal, which was difficult to subtract adequately during image processing, and the intended amplification was not achieved. Variation of the DAB/hydrogen peroxide concentration ratio showed that the mixture containing 0.05% DAB and 0.015% H_2_O_2_ in PBS, pH 7.2 is optimal (Table S1, solution 10). Its application resulted in some coloration of the whole membrane, but it was steady along the strip and reproducible for the assay repetitions, and could be subtracted without compromising the accuracy of the assay results.

**Figure 6:**
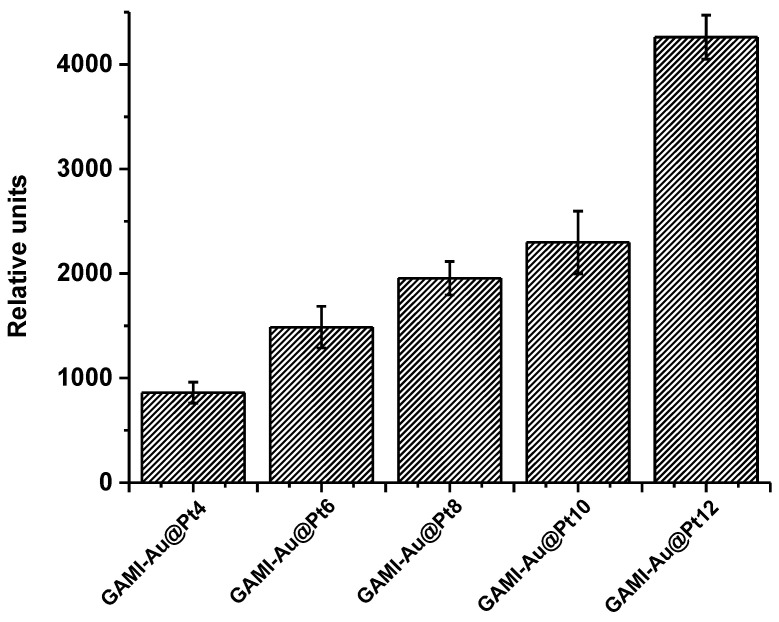
The dependence of signal intensity in the T zone on the loading of GAMI in GAMI–Au@Pt conjugates in the enhanced LFIA of OA. In the control experiment (GAMI loading is 0 µg/mL), there is no analytical signal in the T zone.

The catalytic increase of the signal enabled variations in testing conditions. In particular, the concentration of anti-OA MAbs could be reduced to improve the assay sensitivity. As described above, in the standard LFIA of OA with Au@Pt label, MAbs were added to the tested sample at a concentration of 112.5 ng/mL, and the GAMI–Au@Pt6 conjugate was applied. In the enhanced LFIA, MAbs’ concentration was reduced almost twice (down to 60 ng/mL), and the same labeled conjugate was used. This led to a significant decrease in the colorimetric signal, even under conditions of catalytic amplification (down to 1300 RU), which negatively affected the accuracy and reproducibility of the LFIA. Therefore, selecting the GAMI–Au@Pt conjugate for the enhanced LFIA was necessary. It was shown that upon application of the GAMI–Au@Pt2 conjugate and at MAbs’ concentration of 60 ng/mL, practically no coloration was observed in the T and C zones. So this label was excluded from further consideration. For the remaining conjugates, the signal values in T zones at MAbs’ concentrations of 60 ng/mL are shown in Figure 6.

**Figure 7:**
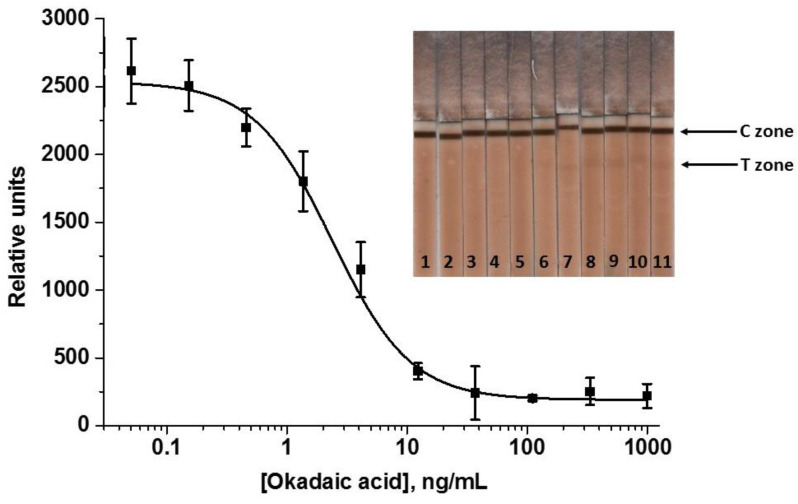
Calibration curve of OA in the enhanced LFIA and images of the test strips. The following OA concentrations were detected (ng/mL): 1000 (1), 333 (2), 111 (3), 37 (4), 12.3 (5), 4.1 (6), 1.4 (7), 0.45 (8), 0.15 (9), 0.05 (10), 0 (11) (*n* = 3).

The obtained data demonstrate that an increase in the GAMI content in the conjugate allowed for the growth in the detected signal up to ~5 times. The maximum detectable signal was achieved using the conjugate with the maximum antibody load—GAMI–Au@Pt12 (~4000 RU). Therefore, this conjugate was selected for the enhanced LFIA of OA, and the concentration of specific MAbs was further reduced to reach a signal comparable to that in the standard LFIA with Au@Pt (~2500 RU). This was achieved at MAbs’ concentration of 45 ng/mL. All other assay conditions (reagents’ concentrations and durations of stages) were the same. The resultant calibration curve of OA and images of scanned test strips after the enhanced LFIA are presented in Figure 7.

A thorough selection of the assay conditions allowed for achieving the OA LOD of 0.5 ng/mL and the cutoff of 10 ng/mL. The range of detectable concentrations was 0.8–6.8 ng/mL. Therefore, the catalytic signal amplification allows for a noticeable gain in the analytical performance of the LFIA: the LOD was decreased by 3 times and the cutoff by 2 times. The assay duration increased only by 2 min (the time for catalytic reaction with DAB).

### 3.4. LFIA of OA in Seawater and Seafood

The developed enhanced LFIA was tested for the determination of OA in real samples, which included seawater, river water, and fish (trout) samples. The sample preparation was developed in our previous studies [19,20,21]. The absence of OA in the tested samples (before spiking) was confirmed by the ELISA using OA ELISA kits (EuroProxima, Arnhem, The Netherlands). According to the proposed methods, for water samples, the matrix effect was eliminated if Triton X-100 (0.05%) was added to water samples, and then the obtained mixtures were diluted 10 times with PBST. For trout, methanol-water extraction of the homogenized fish sample was applied. Several concentrations of OA from the assay’s working range were tested by the enhanced LFIA. The recovery values are presented in Table 2.

According to the data presented in Table 2, the recoveries of OA differed from 89.3 to 123%. The obtained OA values were compared with those estimated in the colorimetric LFIA with AuNPs as a marker. In the latter case, 86.9–126% of OA can be revealed in water and fish samples. Therefore, these test systems can detect OA in comparable quantities. The assay validation was performed using the ELISA kit mentioned above. It was shown that the LFIA/ELISA correlation coefficients for the revealed amounts of OA were high (0.983, *n* = 10).

### 3.5. Advantages of the Proposed LFIA

Increasing sensitivity is a current trend in developing assays for toxic contaminants. This happens not only because of increasingly stringent regulations for toxicants and, consequently, increasing requirements for the quality and environmental friendliness of consumed products, but also due to the peculiarities of sample processing before analysis. The multicomponent composition of the food and water matrix often requires multiple dilutions of samples before testing, which leads to a decrease in the concentration of detected analytes. Therefore, test systems with low LODs are in high demand. Among the approaches used to reduce LODs, new marker systems and assay conditions that ensure the concentrating of labels in the analytical zones of test strips should be noted [37].

In all previous studies of other authors, direct labeling of specific antibodies and the traditional AuNPs label was applied [15,16,17,18]. The assay sensitivity varied from 0.1 to 100 ng/mL for LODs and from 1 to 50 ng/mL for cutoffs. In this study, a combination of the indirect LFIA and a catalytic nanozyme label was proposed to detect aquatic toxin OA for the first time. Earlier, only one immunoanalytical development with the nanozyme label for OA detection had been published. In that work, another kind of immunoassay, namely ELISA, was implemented, which needed laboratory equipment for its completion. Tian et al. (2021) developed a competitive indirect ELISA of OA in shellfish with the Au@Pt NPs/horseradish peroxidase label [38]. ELISA typically has lower LODs than LFIA, and applying the nanozyme led to a further increase in sensitivity; the LOD of OA was 40 pg/mL.

In this study, the use of nanozyme facilitated the achievement of high LFIA sensitivity—0.5 ng/mL for instrumental detection and 10 ng/mL—for the detection by the naked eye, which far exceeds the MRL of OA. In our previous work devoted to developing the enhanced LFIA of OA, better sensitivity was attained using cascade signal amplification. However, 43 min was required only for the analytical procedure without sample preparation. Here, the total testing time from getting a sample to evaluating the results was 25 and 40 min for water and fish, respectively (including 20 min for the LFIA and 5/20 min for water/fish sample preparation). Such high rapidity contributes to the suitability of this test system for mass screening. Other advantages of the LFIA developed in this work include a small volume of the tested sample (50 µL) and an extremely low consumption of specific antibodies (MAbs concentration in the tested sample is only a few tens of ng/mL). The versatility of the anti-species antibodies—nanozyme conjugate allows using it to determine various target compounds without labeling specific antibodies. In this study, the assay was applied both for seafood and water testing. The developed method can be used at any stage of food production—from catching to the manufacture and retail sale of food products.

## 4. Conclusions

An immunochromatographic test system was proposed to determine OA based on nanozyme-based signal amplification. Anti-OA MAbs were produced and tested by ELISA. The Okad D6 clone, which ensured the best assay sensitivity, was chosen for the LFIA development. Au@Pt nanozyme was synthesized and used to label anti-species antibodies. The catalytic properties of the label allowed using it as the peroxidase mimic to enhance the analytical signal by the colorimetric reaction with the peroxidase substrate. The LFIA of OA was carried out in the competitive indirect format. The cutoff/LOD/linear range of the LFIA was 10/0.5/0.8–6.8 ng/mL; the assay duration was 20 min. The sensitivity of the enhanced LFIA was several times higher than that in the assay based on the colorimetric properties of the label (both AuNPs and Au@Pt). The LFIA was efficiently used to detect OA in seawater, river water, and fish (trout) with recoveries of 89.3–123%. Good analytical performance, operating simplicity, and rapidity make this method useful for detecting various toxins and contaminants in food and water.

## Figures and Tables

**Table 1:** Titers of anti-OA MAbs and LODs of OA in the ELISA.

Clone of Anti-OA MAbs	Ig Sub-Isotype	Mab Titer in the ELISA, ng/mL		OA LOD in the ELISA, ng/mL	
		OA–BSA ^1^	OA–STI ^2^	OA–BSA ^1^	OA–STI ^2^
Okad H1	IgG2b	2.5	1.8	0.9	>10,000
Okad B4	IgG2a	0.7	0.5	1.0	3.3
Okad C2	IgG2a	4.2	0.5	1.2	8
Okad D6	IgG2a	6.5	8.7	0.2	0.9

^1,2^ conjugates immobilized on the microplate.

**Table 2:** Recoveries of OA from water and fish samples.

Added OA (ng/mL for Water, µg/g Fir Fish)	Measured OA ± SD (ng/mL for Water, µg/g Fir Fish)	Recovery ± SD ^1^ (%)
Seawater (the Aegean Sea)		
5	5.67 ± 0.005	113.4 ± 0.1
3	3.07 ± 0.23	102.3 ± 7.8
1	0.92 ± 0.042	91.6 ± 4.2
Seawater (the Barents Sea)		
5	4.63 ± 0.26	92.6 ± 5.1
3	3.20 ± 0.31	106.6 ± 10.2
1	0.92 ± 0.05	91.5 ± 4.6
River water (the Volkhov river)		
5	4.65 ± 0.19	93.1 ± 3.8
3	3.48 ± 0.29	115.9 ± 9.8
1	0.89 ± 0.07	89.3 ± 7.4
Fish (trout)		
1.2	1.48 ± 0.13	123.0 ± 10.6
0.8	0.92 ± 0.08	115.4 ± 9.9
0.4	0.41 ± 0.04	101.8 ± 11.1

^1^ Standard deviation, *n* = 3.

## Data Availability

The data supporting the findings of this study are available from the corresponding author upon request.

## Supplementary Materials

The following supporting information can be downloaded at: https://www.mdpi.com/article/10.3390/bios12121137/s1, Figure S1: Dependences of OD_450_ on MAb concentrations in the ELISA with immobilized OA–STI (**a**) and OA–BSA (**b**) for clones Okad H1 (1), Okad B4 (2), Okad C2 (3), and Okad D6 (4).; Figure S2: Calibration curves of OA in the ELISA with immobilized OA–STI (**a**) and OA–BSA (**b**) for clones Okad H1 (1) Okad B4 (2), Okad C2 (3), and Okad D6 (4).; Figure S3: ASM images of AuNPs (**a**) and Au@Pt nanozyme (**b**).; Figure S4: Dependence of the hydrodynamic diameter of Au@Pt nanozyme on Na_2_PtCl_6_ concentration in the reaction mixture measured by DLS; Figure S5: Calibration curve of OA in the standard LFIA with AuNPs and the images of the test strips. The following OA concentrations were detected (ng/mL): 1000 (1), 333 (2), 111 (3), 37 (4), 12.3 (5), 4.1 (6), 1.4 (7), 0.45 (8), 0.15 (9), 0.05 (10); Table S1: Composition of the substrates used in the study and the results of their application in the enhanced LFIA.
