# Supplementary material for: Application of Au@Pt Nanozyme as Enhancing Label for the Sensitive Lateral Flow Immunoassay of Okadaic Acid
Source: Biosensors (Basel). 2022 Dec 7;12(12):1137. doi: 10.3390/bios12121137 (PMC9775009; doi:10.3390/bios12121137)
Supplement: Supplementary file 1 [file biosensors-12-01137-s001.zip › biosensors-2045449-supplementary.pdf]

biosensors

MDPI

Supporting Information

Application of Au@Pt Nanozyme as Enhancing Label for the Sensitive Lateral Flow Immunoassay of Okadaic Acid

Olga D. Hendrickson [1], Elena A. Zvereva [1], Vasily G. Panferov [1], Olga N. Solopova [2], Anatoly V. Zherdev [1], Peter G. Sveshnikov [3], and Boris B. Dzantiev [1,*]

[1] Bach Institute of Biochemistry, Research Center of Biotechnology of the Russian Academy of Sciences, Leninsky Prospect 33, 119071 Moscow, Russia
[2] Blokhin National Medical Research Center of Oncology, Ministry of Health of the Russian Federation, Kashirskoye Shosse 24, 115478 Moscow, Russia
[3] Russian Research Center for Molecular Diagnostics and Therapy, Sympheropolsky Blvrd., 8, 117638 Moscow, Russia
* Correspondence: dzantiev@inbi.ras.ru; Tel.: +7-4959543142

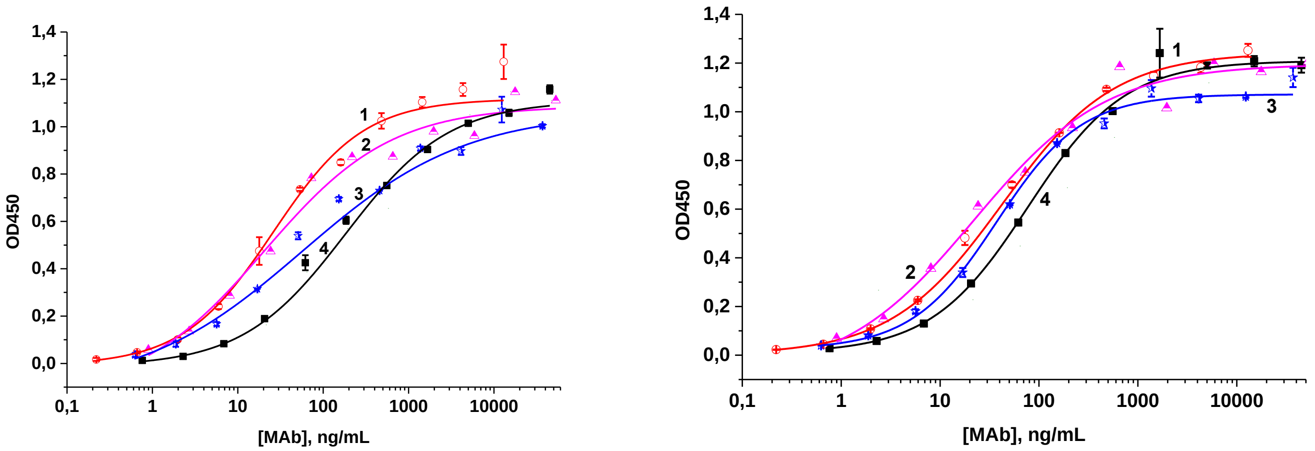

(**a**) (**b**)

Figure S1. Dependences of OD_{450} on MAb concentrations in the ELISA with immobilized OA–STI (**a**) and OA–BSA (**b**) for clones Okad H1 (1), Okad B4 (2), Okad C2 (3), and Okad D6 (4).

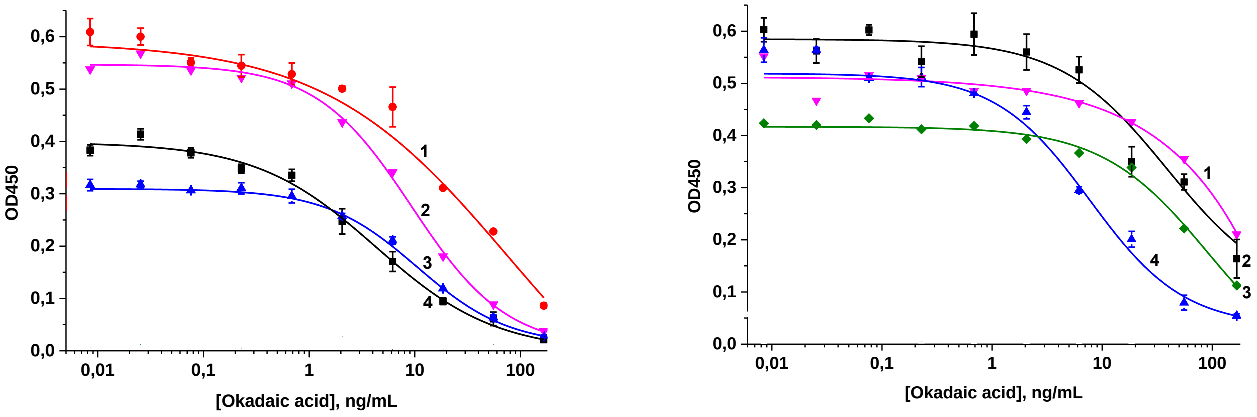

(**a**) (**b**)

Figure S2. Calibration curves of OA in the ELISA with immobilized OA–STI (**a**) and OA–BSA (**b**) for clones Okad H1 (1) Okad B4 (2), Okad C2 (3), and Okad D6 (4).

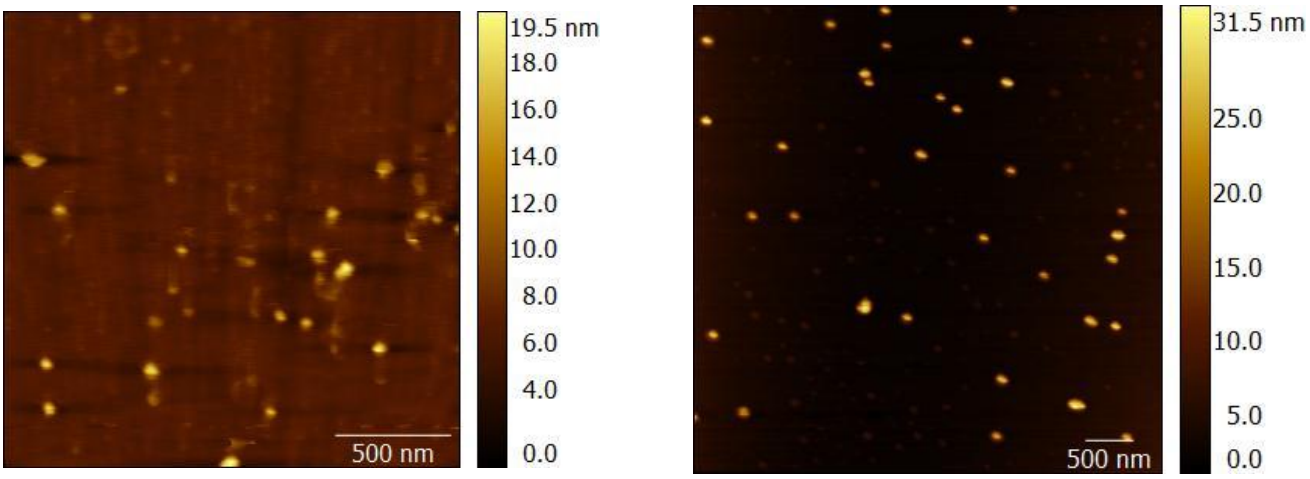

(**a**) (**b**)

Figure S3. ASM images of AuNPs (**a**) and Au@Pt nanozyme (**b**).

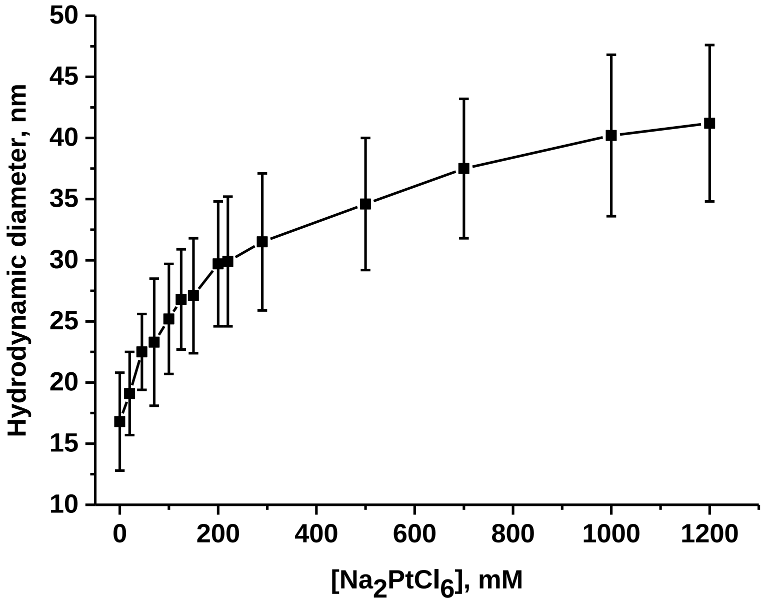

Figure S4. Dependence of the hydrodynamic diameter of Au@Pt nanozyme on Na_2PtCl_6 concentration in the reaction mixture measured by DLS.

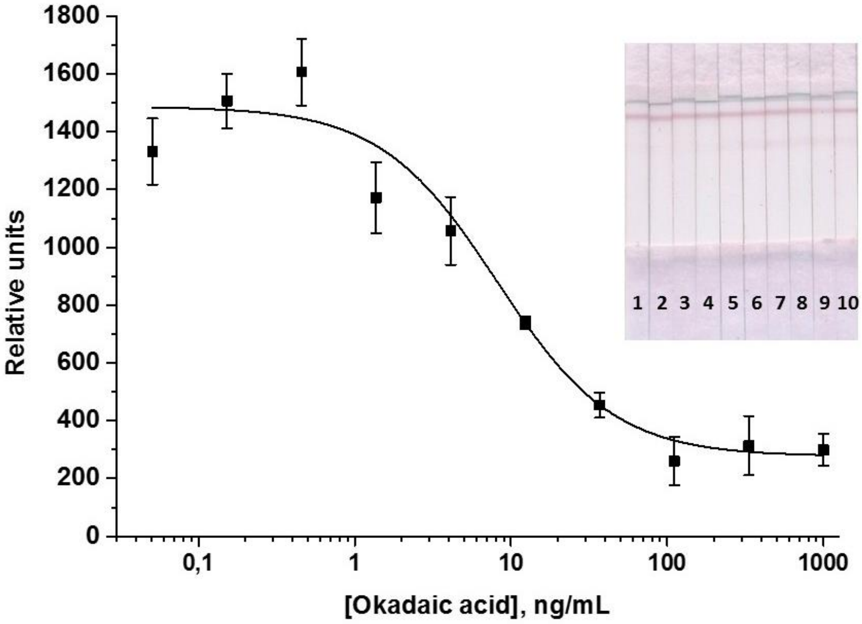

Figure S5. Calibration curve of OA in the standard LFIA with AuNPs and the images of the test strips. The following OA concentrations were detected (ng/mL): 1000 (1), 333 (2), 111 (3), 37 (4), 12.3 (5), 4.1 (6), 1.4 (7), 0.45 (8), 0.15 (9), 0.05 (10).

Table S1. Composition of the substrates used in the study and the results of their application in the enhanced LFIA.

N	Substrate Composition	Signal Amplification	Background in the Zero Point (with No Specific MAbs)	Background Coloration of the Working Membrane
1	TMB-based commercial substrate mixture	yes	yes	Strong, uneven
2	0.05% DAB, 0.02% $CoCl_2{\cdot}6H_2O$, 0.03% H_2O_2 in PBS, pH 7.4	yes	no	Strong, uneven
3	0.05% DAB, 0.02% $CoCl_2{\cdot}6H_2O$, 0.06% H_2O_2 in PBS, pH 7.4	yes	no	Strong, uneven
4	0.05% DAB, 0.01% $CoCl_2{\cdot}6H_2O$, 0.03% H_2O_2 in PBS, pH 7.4	yes	no	Strong, uneven
5	0.05% DAB, 0.03% H_2O_2 in PBS, pH 7.4	yes	no	Average, uneven
6	0.05% DAB, 0.05% $NiCl_2$, 0.7% H_2O_2 in PBS, pH 7.2	yes	no	Strong, uneven
7	0.05% DAB, 0.03% $NiCl_2$, 0.7% H_2O_2 in PBS, pH 7.2	yes	no	Strong, uneven
8	0.05% DAB, 0.02% $NiCl_2$, 0.7% H_2O_2 in PBS, pH 7.2	yes	no	Strong, uneven
9	0.05% DAB, 0.01% $NiCl_2$, 0.7% H_2O_2 in PBS, pH 7.2	yes	no	Average, uneven
10	0.05% DAB, 0.015% H_2O_2 in PBS, pH 7.2	yes	no	Average, even
